# Application of an ultrasound semi-quantitative assessment in the degradation of silk fibroin scaffolds in vivo

**DOI:** 10.1186/s12938-021-00887-3

**Published:** 2021-05-18

**Authors:** Lihui Cai, Nan Gao, TingYu Sun, Ke Bi, Xin Chen, Xia Zhao

**Affiliations:** 1 Department of Otorhinolaryngology-Head and Neck Surgery, Huashan Hospital, Fudan University, 12 Middle Wulumuqi Road, Shanghai, 200040 China; 2 Department of Ultrasound, Shanghai Pulmonary Hospital, Tongji University School of Medicine, 507 Zhengmin Road, Shanghai, 200433 China; 3 State Key Laboratory of Molecular Engineering of Polymers, Department of Macromolecular Science, Laboratory of Advanced Materials, Fudan University, 220 Handan Road, Shanghai, 200433 People’s Republic of China

**Keywords:** Silk fibroin, Ultrasound, Biodegradation, Porous scaffold, In vivo

## Abstract

**Background:**

Research on the degradation of silk fibroin (SF) scaffolds in vivo lacks uniform and effective standards and experimental evaluation methods. This study aims to evaluate the application of ultrasound in assessing the degradation of SF scaffolds.

**Methods:**

Two groups of three-dimensional regenerated SF scaffolds (3D RSFs) were implanted subcutaneously into the backs of Sprague-Dawley rats. B-mode ultrasound and hematoxylin and eosin (HE) staining were performed on days 3, 7, 14, 28, 56, 84, 112, 140, and 196. The cross-sectional areas for two groups of 3D RSFs that were obtained using these methods were semi-quantitatively analyzed and compared to evaluate the biodegradation of the implanted RSFs.

**Results:**

The 3D RSFs in the SF-A group were wholly degraded at the 28th week after implantation. In contrast, the 3D RSFs in the SF-B group were completely degraded at the 16th week. Ultrasonic examination showed that the echoes of 3D RSFs in both groups gradually decreased with the increase of the implantation time. In the early stages of degradation, the echoes of the samples were higher than the echo of the muscle. In the middle of degeneration, the echoes were equal to the echo of the muscle. In the later stage, the echoes of the samples were lower than that of the muscle. The above changes in the SF-B group were earlier than those in the SF-A group. Semi-quantitative analysis of the cross-sectional areas detected using B-mode ultrasound revealed that the degradations of the two 3D RSF groups were significantly different. The degradation rate of the SF-B group was found to be higher than that of the SF-A group. This was consistent with the semi-quantitative detection results for HE staining. Regression analysis showed that the results of the B-mode ultrasound and HE staining were correlated in both groups, indicating that B-mode ultrasound is a reliable method to evaluate the SF scaffold degradation in vivo.

**Conclusions:**

This study suggests that B-mode ultrasound can clearly display the implanted SF scaffolds non-invasively and monitor the degradation of the different SF scaffolds after implantation in living organisms in real-time.

## Background

Silk fibroin (SF) demonstrates significant potential in tissue engineering and biomedical applications owing to its significant biocompatibility, good mechanical properties, and ability to control its biodegradation [1]. SF materials have been processed in various forms, including hydrogels, fibers, microspheres, sponge, microneedles, and three-dimensional regenerated silk fibroin scaffolds (3D RSFs) [2–8]. Different forms of SF have different biological properties, and this applies to degradability. The ideal implant material requires its degradation rate to match the tissue repair rate at the implant site. The biodegradability of the SF materials is one of the criteria to determine whether it can be used as a substitute for tissues.

There is still a lack of uniform and effective standards and test evaluation methods to evaluate the biodegradability of SF materials. For the in vivo evaluation of biological materials, descriptive observations from histological sections are currently used [9–12], which are qualitative methods. In addition, most of the methods that are currently used for degrading SF materials involve invasive tests [13, 14], which are not suitable for living organisms. In order to dynamically evaluate the degradation of SF materials in living organisms, we need to determine a non-invasive, in situ, quantitative, and cost-effective method. Ultrasound, which is a non-invasive technique that can visualize internal structures, has been reported as a method to assess the degradation of biological materials [15]. The principle of ultrasonic detection is that ultrasonic waves are emitted by the generator in the probe and enter the target. After multiple reflections and refractions, they are captured by the receiver in the ultrasonic probe. The ultrasonic diagnostic apparatus can determine the echo intensity of each point in the plane and displayed it on the screen in the form of a gray image, which is the most commonly used brightness mode (B-mode) ultrasonic imaging mode [16]. Recently, some scholars have applied ultrasound to detect SF gels [17]. They tested the degradation process and tissue regeneration (e.g., vascular regeneration) of implanted biomaterials in Wistar rats on the basis of B-mode ultrasound in combination with ultrasound contrast technology. They discovered the value of ultrasound in terms of detecting implanted materials in vivo.

In order to evaluate the biodegradability and biocompatibility of 3D RSFs non-invasively and quantitatively in living organisms, this study used B-mode ultrasound to continuously detect a 3D RSF that was implanted in Sprague-Dawley (SD) rats. These results were compared with those that used hematoxylin and eosin (HE) staining for the first time. Two types of 3D RSFs (SF-A group and SF-B group) were implanted subcutaneously into the back of SD rats. B-mode ultrasound and HE staining were used to measure the cross-sectional areas of the two groups of 3D RSFs, and a statistical analysis was performed. By comparing the detection effects of the two methods on the degradation of the SF scaffolds, the application value of the ultrasound assessment for the degradation of the SF scaffolds was evaluated.

## Results

### General observation

After the subcutaneous implantation of the two groups of 3D RSFs on the back of the SD rats, it was observed that both groups of rats adapted well. In addition, there were no obvious abnormalities in their behavior or diet. The skin surface at the implantation site was slightly elevated and it could be clearly observed in the early stages. The wound showed no signs of redness or infection, and no obvious systemic reactions were found.

The changes in size and the surrounding inflammation of the samples were observed at the implantation site on days 3, 7, 14, 28, 56, 84, 112, 140, and 196 after surgery. In the SF-B group, two samples disappeared on the 84th day (the 12th week) after implantation, and no samples from the SF-B group were found under the skin on the 112th day (the 16th week) after implantation. Therefore, we chose three time points to compare the two groups of the RSF samples: the first week after the operation, the fourth week after the operation, and the 12th week after the operation.

**Fig. 1:**
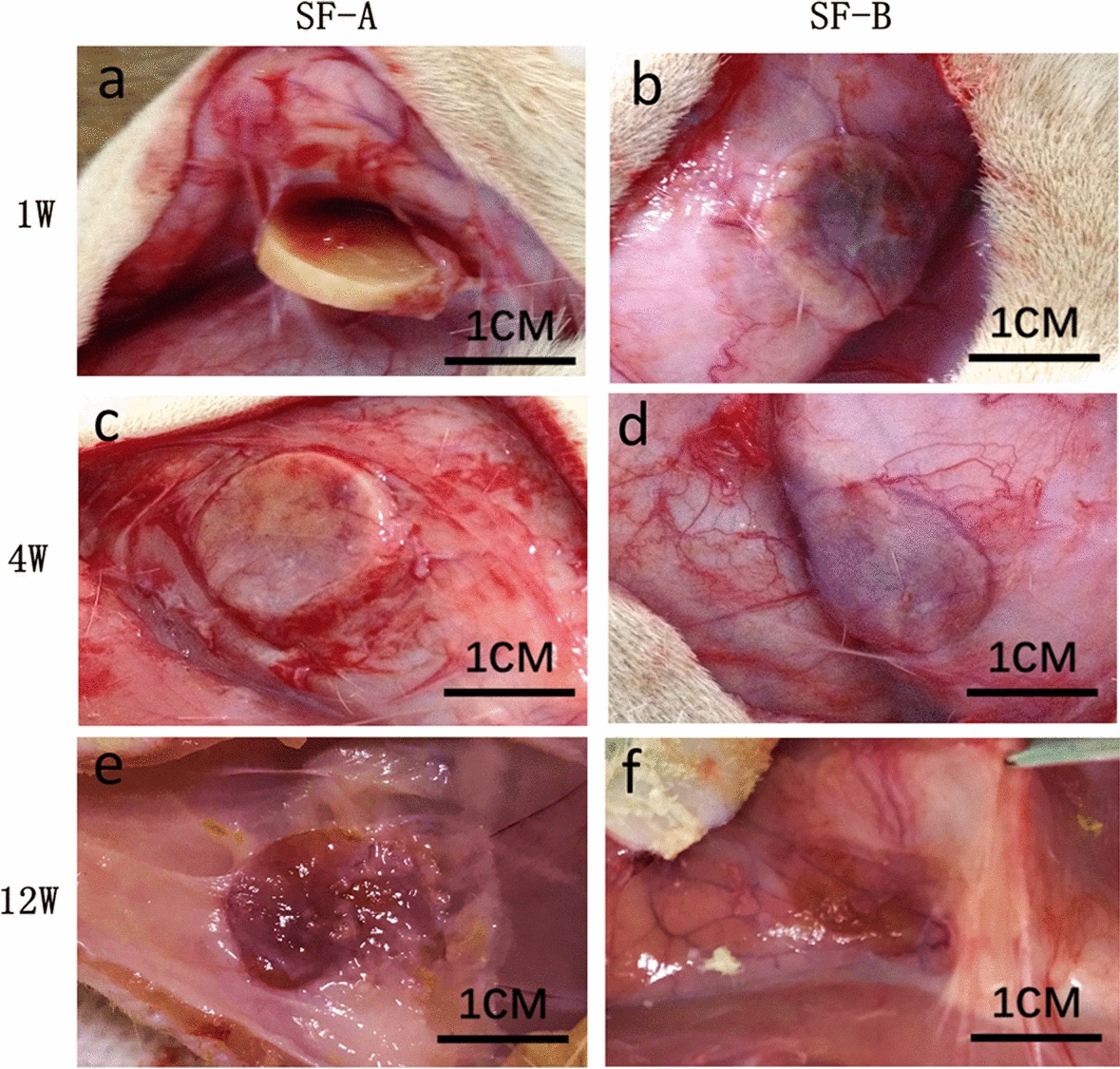
General observation of two types of silk fibroin scaffolds. In situ observation of silk fibroin scaffolds in SF-A group (**a**, **c**, **e**) and SF-B group (**b**, **d**, **f**) at 1, 4, and 12 weeks after implantation

In the first week after implantation, the changes in size of the stent scaffolds at the implantation site and the surrounding tissue reactions were observed. Both of the RSF samples were covered with a thin, translucent fascia-like tissue, and there was no obvious inflammation or granulation formation in the surrounding tissues. The SF-A group sample had no obvious change in the appearance, and the surface was easily separated from the surrounding tissue. The edge of the SF-B group sample was slightly rounded and blunt, and adhered to the surrounding tissue (Fig. 1a, b). During the fourth week after implantation, the surfaces of the two groups of the samples were wrapped with fiber membranes, which were not easily separated from the samples. The edges of the SF-A group samples were slightly rounded and blunt, and the diameter and thickness of the samples rarely changed. The surface fiber membrane of the SF-B group sample was slightly thicker than that of the SF-A, but the overall thickness decreased and the diameter change was not obvious (Fig. 1c, d). During the 12^th^ week after implantation, the overall thickness of the SF-A group sample decreased, but the diameter change was not obvious. The sample in the SF-B group was significantly degraded. Only a pale pink flake tissue remained with a thickness of approximately 1 mm, and the surrounding tissue structure was normal without inflammation (Fig. 1e, f).

Generally, there was no obvious inflammatory response in the surrounding tissues for the two groups of samples during the entire 28-week observation period. The samples in the SF-A group were slightly degraded. By 28 weeks, the volume of the sample and surrounding tissue envelope had fallen to less than one-fifth of the original volume. The samples in the SF-B group rapidly degraded. During the 12th week, only sheet-like tissues with a thickness of about 1 mm remained. No trace of the implant was discovered.

### Examination of the ultrasound morphology

**Fig. 2:**
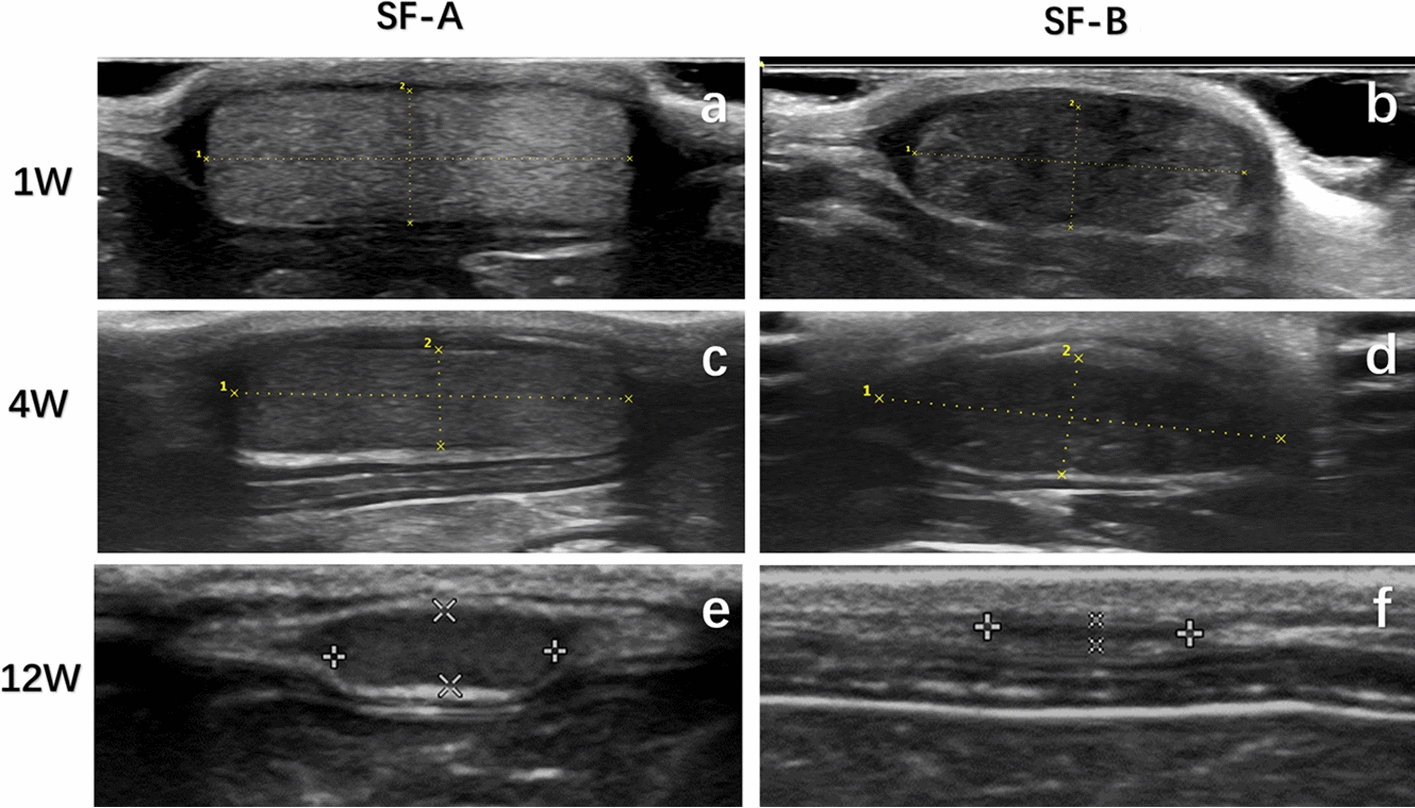
Examination of B-mode ultrasound of two types of silk fibroin scaffolds. B-mode ultrasound examination of silk fibroin scaffolds in SF-A group (**a**, **c**, **e**) and SF-B group (**b**, **d**, **f**) at 1, 4, and 12 weeks after implantation

After the SD rats were anesthetized, the two groups of 3D RSF samples were examined in situ by performing B-mode ultrasound with a high-frequency probe. During the first week after implantation, both groups of samples exhibited isoechoic signals between the subcutaneous layer and the muscle layer. The SF-A group had a clear scaffold boundary and an obvious space-occupying effect. The effusion around the scaffold was slightly more than that in the SF-B group, and there was no obvious hypoechoic inside (Fig. 2a). Most of the scaffolds in the SF-B group had clear boundaries and slightly rounded edges. There was a small amount of mesh-like low echo inside the scaffold and effusion around the scaffold (Fig. 2b). During the fourth week after implantation, the boundary of the SF-A group sample could still be distinguished, and the volume change of the sample was not obvious. The exudation around the sample was absorbed, and the internal echo of the sample was slightly less than one week (Fig. 2c). In the SF-B group, the boundary of the sample was fuzzy, the volume was slightly reduced, and the sample was oval. The internal echo of the sample was lower than that at one week, and the internal mesh-like hypoechoic in the sample gradually increased. The effusion around the sample was also absorbed (Fig. 2d). During the 12th week after implantation, the boundary of the SF-A group sample was blurred and uneven. The volume of the SF-A group sample became smaller than the volume at four weeks, and a sieve-like hypoechoic appeared in the samples. The outer edge of the sample tended to be rounded and blunt, and the internal echo was lower than the echo at four weeks (Fig. 2e). The boundary of the SF-B group sample was difficult to distinguish, and the volume of the sample was significantly reduced in comparison to that at 4 weeks. The internal echo of the SF-B group sample was further reduced, and the mesh-like hypoechoic echoes were scattered throughout the sample (Fig. 2f).

During a total observation period of 28 weeks, the volumes of the two samples gradually decreased and the edges of the samples gradually became blurred, which was difficult to distinguish in the later stage. The echoes of the samples gradually decreased. In the early stages of the degradation, the echo of the sample was higher than that of the muscle. In the middle of the degradation, the echo was equal to that of the muscle. In the later stage, the echo of the sample was lower than that of the muscle. The above-mentioned changes in the SF-B group were earlier than those in the SF-A group. At the 16th week after the silk fibroin scaffolds of the SF-B group were implanted in vivo, we could not find the implanted materials at the implantation site. It meant that the scaffolds of the SF-B group had been completely degraded, and only normal subcutaneous tissue was detected. Therefore, there is no ultrasound data for silk fibroin scaffolds at week 16. The silk fibroin scaffolds in the SF-A group were wholly degraded at the 28th week after implantation in vivo.

### HE staining histological examination

It was observed that the eosinophilic staining of the RSF scaffold appeared as a pink network structure on the HE image. In this experiment, HE staining was used to detect the size change and tissue response of the RSF scaffold that was implanted in vivo.

**Fig. 3:**
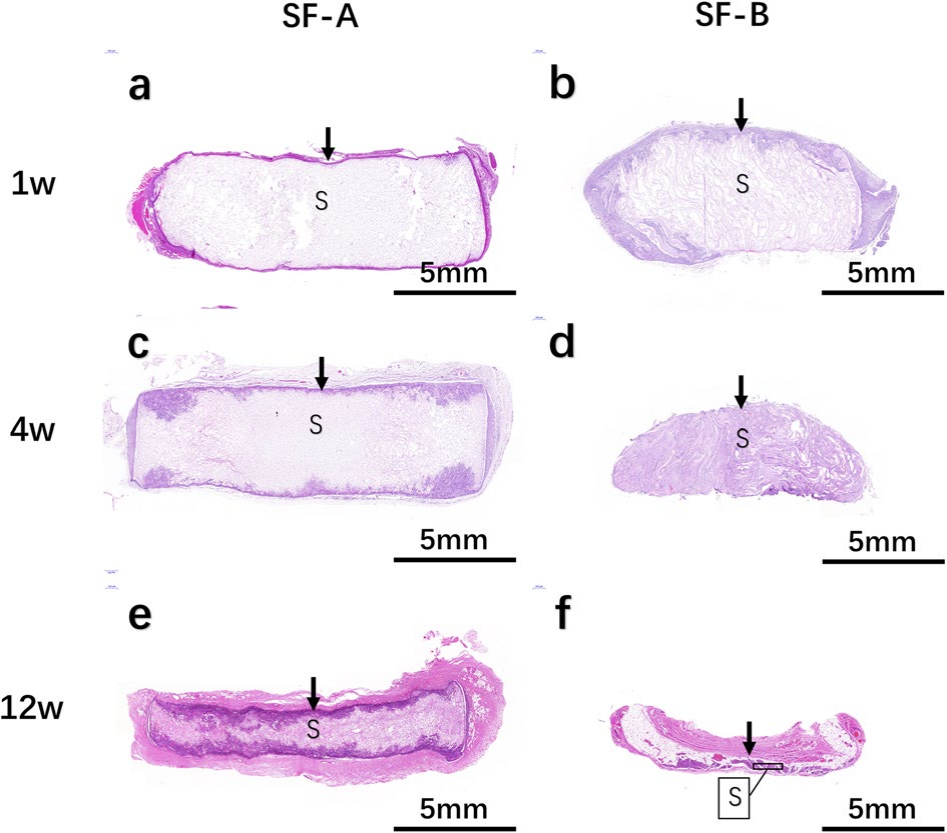
HE staining of two types of silk fibroin scaffolds. HE staining of silk fibroin scaffolds in SF-A group (**a**, **c**, **e**) and SF-B group (**b**, **d**, **f**) at 1, 4, and 12 weeks after implantation. The black arrow points to the junction of material and organization. “S” denotes the scaffold remnant

During the first week after implantation, there was no significant change in the shape and volume of the samples in the SF-A group. The scaffold structure was not significantly damaged. Several layers of fibrocytes, which were arranged in an orderly manner, were observed on the surface of the scaffold. The surrounding tissues were more vascularized and accompanied by inflammatory cell infiltration (Fig. 3a). The samples in the SF-B group were slightly deformed. The edge of the scaffold was slightly rounded and blunt, but the volume change was not obvious. Fibrous tissues were seen around the scaffold, and a large number of inflammatory cells and small blood vessels could be observed. In the tissue–scaffold junction area, there were several fibroblasts that extended inward along the pores, accompanied by lymphocyte and macrophage infiltration, and occasionally, new small blood vessels could be seen (Fig. 3b).

During the fourth week after implantation, the shape and volume of the samples in the SF-A group did not change significantly. The support structure remained generally intact, but it partially collapsed. Several layers of fibrous cells were around the scaffold, which extended irregularly into the scaffold. There were more blood vessels in the tissue–scaffold junction area, which were accompanied by a large number of lymphocytes as well as macrophage infiltration (Fig. 3c). The shape of the SF-B group significantly changed. The edge of the sample was round and blunt, and the volume was slightly smaller. The internal stent collapsed and broke, and the internal void was completely occupied by fibrous tissue, and the broken stent was wrapped around it. We observed a few inflammatory cells, macrophages, and small blood vessels inside the scaffold. A small amount of fibrous tissue was observed around the scaffold (Fig. 3d).

During the 12th week after implantation, the volume of the SF-A group sample was reduced. The supporting structure partially collapsed. The fiber envelope on the surface of the scaffold was further thickened, and a large amount of blood vessels was observed. The fiber cells in the junction area further extended irregularly into the scaffold. Part of the scaffold was broken and fused, and scattered lymphocytes and some macrophages were observed (Fig. 3e). The volume of the sample in the SF-B group was significantly reduced and almost completely degraded. The stent was further broken and divided into several pieces. A large amount of adipose tissue was filled with a small amount of fibrous tissue. There were abundant blood vessels in the surrounding fibrous capsule (Fig. 3f).

During the entire 28-week observation period, the two groups of samples showed different degrees of degradation. The degradation rate of the SF-A group sample was slower than that of the SF-B group sample. By 28 weeks, the volume of the SF-A group sample and its surrounding tissue envelope was less than a quarter of its original volume. The sample in the SF-B group degraded rapidly. During the 12-week period, only flake-like tissues in the SF-B group could be observed with a thickness of less than 1 mm remaining, and could not be found at 16 weeks. During the degradation process, fibroblasts, inflammatory cells, and macrophages were more active in the earlier stages of degradation. In the later stages of degradation, fat cells appeared.

### Comparison of two semi-quantitative assessments of the biodegradation of the RSFs

**Fig. 4:**
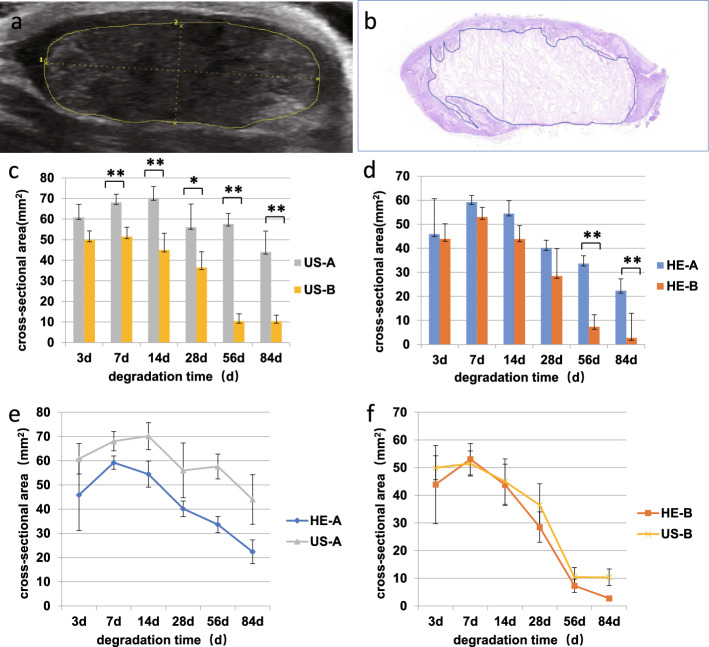
Comparison of two semi-quantitative assessments of the biodegradation of two types of silk fibroin scaffolds. **a** A schematic diagram of the measured area of the material in the ultrasound image: the area circled by the yellow line is the measured area. **b** A schematic diagram of the measured area of the material in the histological examination image: the area circled by the blue line is the measured area. **c** The cross-sectional areas for two groups of SF scaffolds (A and B) detected by B-mode ultrasound (US)after 3, 7, 14, 28, 56, and 84 days of subcutaneous implantation. **d** The cross-sectional areas for the two groups of SF scaffolds (A and B) detected by HE staining (HE) after subcutaneous implantation. **e** The cross-sectional areas of SF-A detected by HE staining (HE) and B-mode ultrasound (US)after 3, 7, 14, 28, 56, and 84 days of subcutaneous implantation. **f** The cross-sectional areas of SF-B detected by HE staining (HE) and B-mode ultrasound (US)after subcutaneous implantation. (*Statistically significant differences at *P* ≤ 0.05; ** Statistically significant differences at *P* ≤ 0.01)

According to the results of the B-mode ultrasound examination, the cross-sectional areas of the two 3D RSFs were significantly different at almost all the time points except on the third day (Fig. 4c). However, according to the results of the histological examination of the HE staining, there was no significant difference in the cross-sectional areas of the two groups at 3, 7, 14, and 28 days after implantation, but there were significant differences on days 56 and 84 (Fig. 4d). The Pearson correlation coefficient was used to correlate the cross-sectional areas of SF-A at each time point that was obtained by ultrasound with the cross-sectional areas of SF-A that were detected by HE staining. The results showed that the cross-sectional areas of SF-A that were obtained by ultrasound were closely related to the cross-sectional areas of SF-A that were obtained by HE staining (*r* = 0.955, *P* < 0.01) (Fig. 4e). The Pearson correlation coefficient was used to correlate the cross-sectional areas of SF-B at each time point that was obtained by ultrasound with the cross-sectional areas of SF-B detected by HE staining. The results showed that the cross-sectional areas of SF-B that were obtained by ultrasound were closely related to the cross-sectional areas of SF-B that were obtained by HE staining (*r* = 0.986, *P* < 0.01) (Fig. 4f).

## Discussion

The literature that addresses the degradation of SF materials in vivo lacks uniform and effective standards and experimental evaluation methods. A common method is the morphological description, which includes HE staining and scanning electron microscopy [13, 18]. The strength of the material was evaluated based on its mechanical properties [19]. The biocompatibility and in vivo absorption of the material were detected through cell biology [14, 20]. Functional indicators could also be tested for the materials that were implanted in special parts of the body [21]. Although these methods have a certain effect on understanding the reaction process and the mechanism of degradation in vivo, it is often impossible to accurately quantify the material degradation dynamically and in real-time. Most of the tests that are described in the literature are invasive tests; however, they are not applicable to living organisms. Although imaging inspection methods such as X-ray inspection and computed tomography inspection are non-invasive [22], their application range is limited and they are only sensitive to denser materials. They are not suitable for the study of SF materials with a lower density. Therefore, finding a dynamic and non-invasive degradation evaluation method is also an urgent problem that needs to be solved in the clinical applications of SF materials.

In a previous study [14], we developed a semi-quantitative approach to assess the in vivo degradation rate and biocompatibility of the 3D RSFs that have different pore sizes. The semi-quantitative method was used to evaluate the biodegradation by measuring the thickness of the residual scaffolds, fibrous capsules, and infiltrated tissues by performing a histological analysis. Since the scaffold’s volume change is irregular after implantation, the comparison method that is based on the thickness of the scaffold cannot accurately reflect the degradation of the scaffold. In fact, the best way to estimate the rate of the scaffold biodegradation is to measure the residual volume of the scaffold in three dimensions. However, this method is difficult to implement in vivo. Wang et al. [19] compared the biodegradation of several scaffolds (5 mm in diameter and 2.5 mm in thickness) by measuring the cross-sectional area of the residual scaffolds in two dimensions. This is a relatively accurate method. However, since this method was technologically limited at that time, only small scaffolds could be measured. In this study, we made improvements based on the previous studies. HE-stained sections were made by selecting the largest cross-section of the scaffold at each time point after the implantation. As long as the size of the scaffold does not exceed the size of the slice of the HE stain, the image on the slice can be completely recorded by the scanning system. A semi-quantitative comparison was performed on the cross-sectional area of the scaffolds to evaluate the degradability of the RSFs. Histological methods can intuitively obtain the changes in size of the scaffolds; thus, evaluating the scaffold degradation is more reliable. However, this method cannot be applied to study scaffold degradation in living organisms. In order to facilitate the research after the scaffold is implanted in living organisms, we urgently need a non-invasive, real-time, repetitive method that is applicable.

Ultrasound is a non-invasive imaging method that is more sensitive to the detection of soft tissues. It has been used in research that involves degrading biological materials. Li et al. [23] investigated the neovascularization and biodegradation of an SF gel in vivo using multiple mode ultrasound by quantifying the echo intensity, volume, and contrast enhancement of the SF gel implants. It showed that the silk gel implants appear as hypoechoic nodules in the early stages of the degradation, and there are clear boundaries under the skin. With the passage of time, the echogenicity of the silk hydrogel implants gradually increased, and it was demonstrated through medium grayscale images. In the late stage of the degradation, the echogenicity of the silk hydrogel implant was equal to that of the adjacent border, and it could not be clearly identified until the 20^th^ day. This study showed that ultrasound might be used to study the degradation of silk fibroin gel in vivo. However, few studies have been published applying ultrasound to other forms of silk fibroin materials (such as fibers and silk fibroin scaffolds, among others). Various forms of silk fibroin materials have different biological properties and degradabilities. 3D RSFs have a wide range of applications in biomedical engineering and tissue engineering. Therefore, a suitable degradation detection method has a positive clinical application value for the in vivo degradation of 3D RSFs. In this study, we applied B-mode ultrasound to investigate the in vivo degradation of RSF scaffolds. With the passage of time, the echoes of the SF scaffold gradually decreased. In the early stages of the degradation, the echo of the sample was higher than that of the muscle. In the middle of the degradation, the echo was equal to the that of the muscle. In the later stage, the echo of the sample was lower than that of the muscle. The echo of the SF scaffold during ultrasound in this study was different from the findings by Li et al. [23]. It may due that various formulations of silk fibroin have different densities and uniformity. The silk fibroin scaffold used in our experiment has a low density and contains more pores, so its uniformity is poor. In contrast, the silk fibroin gel used in Li’s experiment has a relatively high density and is relatively uniform. According to the principle of ultrasound, the echo intensity of ultrasound varies with the acoustic impedance of the material [16]. The acoustic impedance is related to the density and uniformity of the material [24]. Generally speaking, the higher the density or the more uneven the material, the stronger the acoustic impedance and the higher the echo intensity. Therefore, the echo intensity of various forms of SFs under ultrasound is different. In addition, in this experiment, we found that the echoes of the SF scaffolds gradually decreased with the increase of the implantation time. The reason for the changes in the echoes in this study might be related to the degree of tissue cells that invade the scaffolds and the degradation of the scaffolds. During the early stage of implantation, the scaffolds were filled with pores, only a small number of tissue cells invaded the scaffolds, and the scaffolds were hardly degraded. As a result, the echo of the scaffolds was higher than that of the muscle in the early stage. As the degradation progresses, tissue cells invade the scaffolds, and the pores in the scaffolds gradually degrade; therefore, the echo of the scaffolds is reduced. In the later stage, the scaffolds were filled with cells, which form a relatively uniform whole; hence, it appears to be hypoechoic when applying ultrasound. In these experiments, local hypoechoic echoes were observed in the scaffolds. We believe that this may be due to the penetration of the host tissue into the scaffolds and the degradation and absorption of the local scaffolds. In the previous studies of our research group [14], it has been shown that the biodegradation of RSFs is mediated by inflammation to a certain extent. The microporous shape of the scaffold is conducive to the infiltration of inflammatory factors. As the host tissue entered the scaffold, inflammatory factors such as macrophages/multinucleated giant cells were brought into the scaffold. In this study, two groups of silk fibroin scaffolds with different pore sizes were selected. The different pore diameters resulted in different host tissue penetration rates of scaffolds in both groups. Scaffolds with more prominent pores will make them more sensitive to cell invasion and lead to a faster rate of degradation of the scaffold in vivo. Therefore, it can be inferred that the degradation of the SF scaffold and tissue proliferation occur simultaneously. The performance of the tissue deep into the scaffold could also be observed in the HE-stained sections, which further confirmed this idea.

In this study, we implanted two types of 3D RSFs (SF-A group and SF-B group) that were subcutaneously placed into the back of SD rats. For a semi-quantitative comparison, we used B-mode ultrasound and HE staining to measure the cross-sectional areas of two groups of 3D RSFs and performed a statistical analysis. The statistical results show that there were significant differences in the degradation of the two groups of 3D RSFs. The degradation rate of the SF-B group was significantly higher than that of the SF-A group. The difference in the degradation rate of both silk fibroin scaffolds may be mainly related to their pore sizes. Our previous study has shown that the larger the pore size of the silk fibroin scaffold, the faster the degradation rate [14]. The silk fibroin pore size of the SF-B group was larger than that of the SF-A group, so the degradation rate of the SF-B group is higher than that of the SF-A group. Regression analysis showed that the results of the B-mode ultrasound and HE staining were correlated in both groups. This indicates that B-mode ultrasound is reliable for the degradation of the SF scaffolds in vivo. In this study, we determined that the volumes of the two types of 3D RSFs increased after one week of implantation by applying two detection methods. From the preparation method, it can be seen that the obtained SF scaffolds were thoroughly washed with deionized water in order to remove the n-butanol. In addition, after sterilizing in 75% (v/v) ethanol solution, the SF scaffolds were continuously stored in sterilized water for at least one day. All these treatments implied that the SF scaffolds were reached a swelling equilibrium before the implantation. Therefore, it have little possibility that the increase in the volume of scaffolds was due to the swelling of the SF scaffold, so we attributed the possible reason to the appearance of the fibrous capsule around the scaffold after one week of implantation, and the scaffold was not significantly degraded at that time. Therefore, the volume of the scaffold is larger in comparison with when it was implanted. In addition, the infiltration of the inflammatory cells and the penetration of small blood vessels also slightly increased the volume of the scaffold. It can be observed from the figure that the cross-sectional areas of the ultrasound detection are slightly larger than those observed during HE detection (Fig. 4). A possible reason for this deviation is that ultrasound can observe the material in situ when it is placed in the body. After the SF scaffolds are implanted in the body, a fibrous membrane gradually forms on the surface of the SF scaffold. In other words, a material–tissue complex is formed, which shows a similar echo to the scaffold; thus, it can be detected as a scaffold with ultrasound. HE staining was conducted after the materials were removed from the body, and could clearly distinguish the material and the fiber envelope; therefore, the detected value in HE staining was less than that of ultrasound. In addition, dehydration during HE staining of the implanted materials may also lead to a reduction in the volume. In the experiment, it was determined that the material–tissue complex of SF-A was thicker than the material–tissue complex of SF-B. This may be related to the fact that SF-A is denser and does not easily degrade. We believe that the material–tissue complex is a common whole in the body's repair process. In addition, the detection of the material–tissue complex with ultrasound can better reflect the true situation of the repair process. In addition, ultrasound can also detect inflammation and exudation around the material in the early implantation stage, which is not possible with other invasive examinations.

In this study, it was determined that the application of ultrasound to evaluate the degradation of SF scaffolds has some limitations. In the early and middle stages of the degradation process, the SF scaffolds can be clearly detected by applying B-mode ultrasound in vivo. The echo changes accompanying the material degradation and host tissue infiltration can be observed. However, when the material was gradually degraded and absorbed in the later stage of the degradation, the echo was reduced. In other words, when there was not much material left, it was difficult to distinguish the material from the surrounding tissues, and applying ultrasound during the later stages of implantation led to the loss of its advantages. Nevertheless, ultrasound is still a non-invasive and real-time method for biomaterials implanted in the body. Ultrasound is also available for studying the in vivo degradation of other types of biomaterials. In a recent study, ultrasonography was performed to assess the degradation of scaffold-forming collagen implanted in Pig breasts [25]. It can be inferred that ultrasound can be applied when the echo of the material is different from the tissue of the implantation site or there is a gap between the implantation sites. However, it should be noted that the biomaterial may interact with the body after being implanted, and its ultrasonic performance may change with the time of implantation. Therefore, in the application of ultrasonic testing of biomaterials, it is also necessary to conduct comprehensive and long-term observations on the characteristics of the materials.

## Conclusions

Although ultrasound is a commonly used clinical tool, its usage with regard to SF materials has not received significant attention. This study applied B-mode ultrasound to observe and semi-quantitatively assess the degradation of SF scaffolds, and compared it with HE staining. It showed that B-mode ultrasound can clearly display the implanted SF scaffold and monitor the degradation of different SF scaffolds after implantation in living organisms in real-time. Although the size of the SF scaffold measured by ultrasound is larger than that of the HE staining at the same time point, the change in the size of the scaffold that is measured sequentially by applying B-mode ultrasound has a good correlation with HE staining. In addition, as the SF scaffold degrades, its echo gradually decreases. In the early stages of the degradation, the echo of the SF scaffold is higher than that of the muscle. In the middle of the degradation, the echo is equal to that of the muscle. In the later stage, the echo is lower than that of the muscle. Recent studies [23] have shown that the different forms of SF materials (e.g., gels) have different ultrasound performances. Therefore, future studies should focus on determining the ultrasonic performance for the different forms of SF materials. This can provide a comprehensive reference for ultrasound when evaluating the degradation of SF materials in living organisms.

## Methods

### Preparation and measurement of the 3D RSFs

Two types of 3D RSFs with different pore sizes were prepared according to the procedures that were described previously [26, 27]. Briefly, the treated Bombyx mori cocoons were degummed in 0.5 wt% Na_2_CO_3_ solution for 30 min. Then, they were drained and dried in a drying oven at 40 °C for three days after being rinsed thoroughly with deionized water. The degummed silk fibers were dissolved in 9.5 mol/L LiBr solution at 60 °C for 1 h. After filtration, the resulting SF solution was dialyzed for three days and concentrated to 14% w/w with an aqueous polyethylene glycol solution. n-Butanol was added to the SF solution with gentle stirring to form a stable oil/water emulsion. The volume ratios of n-butanol to the mixture solutions were 1:1(SF-A) and 1:100(SF-B). The two mixture solutions were poured into cylindrical molds with a diameter of 13 mm and they were frozen in a refrigerator at −20 °C for 24 h. After thawing, the two SF scaffolds were sliced with a microtome into a flat cylindrical shape with a diameter of approximately 13 mm and a thickness of about 4 mm, which are named SF-A and SF-B, respectively. They were washed thoroughly with deionized water to remove the n-butanol and uncross-linked SF in the RSFs. Before experimental use, the scaffolds were immersed in a 75% (v/v) ethanol solution for sterilization and stored in sterilized water at 4 °C until use.

### Implantation of the 3D RSFs

For this study, 36 male SD rats whose weights were between 150 and 200 g were randomly divided into two groups. Two types of RSFs were implanted by using the experimental procedures that are described in the literature [14, 28]. The rats were anesthetized with an intraperitoneal injection of 10% chloral hydrate (4 mL/kg body weight). Four paravertebral incisions (1.5 cm each) were made on the back of the rats, which were approximately 1.5 cm lateral to the vertebral column. Subcutaneous pockets were formed by blunt dissection. Two kinds of RSFs were randomly implanted into the pockets according to the group, and then the incisions were closed with a 4-0 mousse thread. All the experiments were performed under aseptic conditions.

### Ultrasound in situ

After the SD rats were anesthetized with 2% isoflurane, the back skin of the rats was prepared with hair removal cream. On days 3, 7, 14, 28, 56, 84, 112, 140, and 196, three RSF samples were randomly selected from each group for ultrasound examination. The MyLab twice ultrasound diagnostic instrument (Esaote, Genova, Italy) was used, and it was equipped with a high-frequency linear probe (SL3116, frequency of 18 MHz) in the B-mode to observe the shape and echo intensity of the RSF samples. The scanning depth (2 cm), frequency (22 MHz), and gain settings (62%) were initially optimized and maintained to be constant throughout the imaging. The scanning area contained the scaffold and the surrounding host tissue. In each sample, three cross-sectional images across the center of the sample were selected. The cross-sectional images of the RSF samples at different time points were acquired for the offline analysis.

### Histological analysis

On days 3, 7, 14, 28, 56, 84, 112, 140, and 196 after surgery, two animals from each group were randomly selected and euthanized by applying CO_2_ suffocation, and then eight RSF samples were collected. Three samples were randomly selected for histological evaluation. HE staining was performed to evaluate the biodegradation of the RSFs. In each sample, a cross-section was selected across the center of the sample. The scanner (Pannoramic MIDI, 3Dhistech) and browse software (CaseViewer 2.4) were used to obtain the area of each cross-section.

### Statistical analysis

All the data are expressed as the mean ± standard deviation. The statistical analysis was performed using the SPSS23.0 software program. The cross-sectional areas of SF-A and SF-B at different time points were compared by performing the one-way analysis of variance. The Pearson correlation coefficient was used to correlate the calculated parameters that were derived from HE staining and the ultrasound images. The values of *P* < 0.05(*) and 0.01(**) were considered to be statistically significant.

## Data Availability

The datasets analyzed during the current study are available from the corresponding author on reasonable request.

## Abbreviations

HE: Hematoxylin and eosin
SD: Sprague-Dawley
SF: Silk fibroin

## References

[1] Altman GH, Diaz F, Jakuba C, Calabro T, Horan RL, Chen J, Lu H, Richmond J, Kaplan DL (2003). Silk-based biomaterials. Biomaterials.

[2] Qian KY, Song Y, Yan X, Dong L, Xue J, Xu Y, Wang B, Cao B, Hou Q, Peng W (2020). Injectable ferrimagnetic silk fibroin hydrogel for magnetic hyperthermia ablation of deep tumor. Biomaterials.

[3] Opalkova SA, Kozma E, Opalek A, Kronekova Z, Kleinova A, Nagy S, Kronek J, Rydz J, Eckstein AA (2020). Diclofenac embedded in silk fibroin fibers as a drug delivery system. Materials (Basel).

[4] Radu IC, Biru IE, Damian CM, Ion AC, Iovu H, Tanasa E, Zaharia C, Galateanu B (2019). Grafting versus Crosslinking of Silk Fibroin-g-PNIPAM via Tyrosine-NIPAM Bridges. Molecules.

[5] Gong H, Wang J, Zhang J, Wu J, Zheng Z, Xie X, Kaplan DL, Li G, Wang X (2019). Control of octreotide release from silk fibroin microspheres. Mater Sci Eng C Mater Biol Appl.

[6] Wei W, Liu J, Peng Z, Liang M, Wang Y, Wang X (2020). Gellable silk fibroin-polyethylene sponge for hemostasis. Artif Cells Nanomed Biotechnol.

[7] Zhu M, Liu Y, Jiang F, Cao J, Kundu SC, Lu S (2020). Combined silk fibroin microneedles for insulin delivery. Acs Biomater Sci Eng.

[8] Guan Y, You H, Cai J, Zhang Q, Yan S, You R (2020). Physically crosslinked silk fibroin/hyaluronic acid scaffolds. Carbohydr Polym.

[9] Paula AB, Laranjo M, Marto CM, Paulo S, Abrantes AM, Fernandes B, Casalta-Lopes J, Marques-Ferreira M, Botelho MF, Carrilho E (2019). Evaluation of dentinogenesis inducer biomaterials: an in vivo study. J Appl Oral Sci..

[10] Campos F, Bonhome-Espinosa AB, Chato-Astrain J, Sanchez-Porras D, Garcia-Garcia OD, Carmona R, Lopez-Lopez MT, Alaminos M, Carriel V, Rodriguez IA (2020). Evaluation of fibrin-agarose tissue-like hydrogels biocompatibility for tissue engineering applications. Front Bioeng Biotechnol.

[11] Lindner C, PrOhl A, Abels M (2020). Specialized histological and histomorphometrical analytical methods for biocompatibility testing of biomaterials for maxillofacial surgery in (pre-) clinical studies. Vivo.

[12] Kubikova T, Filova E, Prosecka E, Plencner M, Kralickova M, Tonar Z (2015). Histological evaluation of biomaterials administration in vivo on the cartilage, bone and skin healing. Cas Lek Cesk.

[13] Guan G, Wang L, Li M, Bai L (2014). In vivo biodegradation of porous silk fibroin films implanted beneath the skin and muscle of the rat. Biomed Mater Eng.

[14] Guo Y, Chen Z, Wen J, Jia M, Shao Z, Zhao X (2017). A simple semi-quantitative approach studying the in vivo degradation of regenerated silk fibroin scaffolds with different pore sizes. Mater Sci Eng C Mater Biol Appl.

[15] Wong LC, Chiu WK, Russ M, Liew S (2012). Review of techniques for monitoring the healing fracture of bones for implementation in an internally fixated pelvis. Med Eng Phys.

[16] Abu-Zidan FM, Hefny AF, Corr P (2011). Clinical ultrasound physics. J Emerg Trauma Shock.

[17] Leng X, Liu B, Su B, Liang M, Shi L, Li S, Qu S, Fu X, Liu Y, Yao M (2017). In situ ultrasound imaging of silk hydrogel degradation and neovascularization. J Tissue Eng Regen Med.

[18] Kim JH, Park CH, Lee OJ, Lee JM, Kim JW, Park YH, Ki CS (2012). Preparation and in vivo degradation of controlled biodegradability of electrospun silk fibroin nanofiber mats. J Biomed Mater Res A.

[19] Wang Y, Rudym DD, Walsh A, Abrahamsen L, Kim HJ, Kim HS, Kirker-Head C, Kaplan DL (2008). In vivo degradation of three-dimensional silk fibroin scaffolds. Biomaterials.

[20] Fan H, Liu H, Toh SL, Goh JC (2009). Anterior cruciate ligament regeneration using mesenchymal stem cells and silk scaffold in large animal model. Biomaterials.

[21] Park SH, Gil ES, Kim HJ, Lee K, Kaplan DL (2010). Relationships between degradability of silk scaffolds and osteogenesis. Biomaterials.

[22] Lu S, Wang P, Zhang F, Zhou X, Zuo B, You X, Gao Y, Liu H, Tang H (2015). A novel silk fibroin nanofibrous membrane for guided bone regeneration: a study in rat calvarial defects. Am J Transl Res.

[23] Li S, Yu D, Ji H, Zhao B, Ji L, Leng X (2018). In vivo degradation and neovascularization of silk fibroin implants monitored by multiple modes ultrasound for surgical applications. Biomed Eng Online.

[24] Noce JP (1990). Fundamentals of diagnostic ultrasonography. Biomed Instrum Technol.

[25] Puls TJ, Fisher CS, Cox A, Plantenga JM, McBride EL, Anderson JL, Goergen CJ, Bible M, Moller T, Voytik-Harbin SL (2021). Regenerative tissue filler for breast conserving surgery and other soft tissue restoration and reconstruction needs. Sci Rep.

[26] Cao Z, Wen J, Yao J, Chen X, Ni Y, Shao Z (2013). Facile fabrication of the porous three-dimensional regenerated silk fibroin scaffolds. Mater Sci Eng C Mater Biol Appl.

[27] Wen J, Yao J, Chen X, Shao Z (2018). Silk fibroin acts as a self-emulsifier to prepare hierarchically porous silk fibroin scaffolds through emulsion-ice dual templates. ACS Omega.

[28] Ni Y, Zhao X, Zhou L, Shao Z, Yan W, Chen X, Cao Z, Xue Z, Jiang JJ (2008). Radiologic and histologic characterization of silk fibroin as scaffold coating for rabbit tracheal defect repair. Otolaryngol Head Neck Surg.

